# Molecular mechanisms of collateral sensitivity to the antibiotic nitrofurantoin

**DOI:** 10.1371/journal.pbio.3000612

**Published:** 2020-01-27

**Authors:** Roderich Roemhild, Marius Linkevicius, Dan I. Andersson

**Affiliations:** Department of Medical Biochemistry and Microbiology, Uppsala University, Uppsala, Sweden; Universität zu Köln, GERMANY

## Abstract

Antibiotic resistance increasingly limits the success of antibiotic treatments, and physicians require new ways to achieve efficient treatment despite resistance. Resistance mechanisms against a specific antibiotic class frequently confer increased susceptibility to other antibiotic classes, a phenomenon designated collateral sensitivity (CS). An informed switch of antibiotic may thus enable the efficient treatment of resistant strains. CS occurs in many pathogens, but the mechanisms that generate hypersusceptibility are largely unknown. We identified several molecular mechanisms of CS against the antibiotic nitrofurantoin (NIT). Mutants that are resistant against tigecycline (tetracycline), mecillinam (β-lactam), and protamine (antimicrobial peptide) all show CS against NIT. Their hypersusceptibility is explained by the overexpression of nitroreductase enzymes combined with increased drug uptake rates, or increased drug toxicity. Increased toxicity occurs through interference of the native drug-response system for NIT, the SOS response, with growth. A mechanistic understanding of CS will help to develop drug switches that combat resistance.

## Abstract 2

Resistance mechanisms against a specific antibiotic class frequently often confer negative cross-resistance to other antibiotic classes, a phenomenon known as collateral sensitivity. This study shows that collateral sensitivity in bacteria can be explained by a combination of several mechanisms that can be exploited to develop drug switches that combat resistance.

## Introduction

Antibiotic resistance is a growing problem, and it can emerge during antibiotic therapy as a consequence of gene transfer, or the selection of spontaneous mutants [1–4]. Particularly problematic multiresistant variants of pathogenic bacteria can evolve through consecutive genetic changes or by acquiring, for example, plasmids that carry several resistance determinants. The global spread of resistant and multiresistant strains increasingly obstructs the treatment of bacterial infections [5] and causes an estimated 700,000 deaths annually [6]. There is a critical need to find new ways to achieve efficient treatment despite resistance and to reduce the emergence and long-term persistence of resistance. An Achilles heel of drug resistance has emerged in recent years that potentially works towards both goals, so-called collateral sensitivity (CS) [7,8].

CS is negative cross-resistance to antibiotics and is a common pleiotropic consequence of resistance mutations [7–12] and resistance genes [13]. CS predominantly occurs between antibiotics that have different killing mechanisms. For example, resistance to aminoglycoside antibiotics is frequently associated with increased susceptibility to β-lactam antibiotics in *Escherichia coli* [8,14]. CS usually means an increase in susceptibility by a factor of 2–4×, but there are also stronger effects. Resistant bacteria may thus be efficiently eradicated by switching treatment to a different antibiotic class towards which the pathogen displays CS. It has further been proposed that CS can reduce or prevent the emergence of multidrug resistance. Sequential multidrug treatments that cycle between antibiotics with CS to each other may potentially reduce the rate of resistance evolution, that is, an emerging resistant subpopulation is outcompeted by sensitive cells following a switch of antibiotics that targets their CS. This process could be serially repeated in a sequential multidrug treatment so as to maintain antibiotic susceptibility in the pathogen. A potential utility of such sequential treatments is supported by a growing collection of experimental studies in several species of bacteria [15]. However, we still have little understanding of the molecular mechanisms that lead to CS.

We describe here the molecular mechanisms of CS in *E*. *coli* and *Salmonella enterica*, using a functional genetics approach. We previously observed that spontaneously resistant mutants against several classes of antibiotic produced strong CS to the antibiotic nitrofurantoin (NIT), ranging from an 8× to 35× increase in susceptibility depending on the resistance mutation [16–18]. NIT is a front-line drug for the treatment of uncomplicated urinary tract infections, of which *E*. *coli* is the primary cause. The drug has multiple mechanisms of action that are poorly understood but involve damage to DNA [19,20] and ribosomes [21]. To exert its bactericidal activity, NIT has to be enzymatically activated in the cell, which occurs through the activity of nitroreductases [22], of which NfsA and NfsB are the major enzymes. Because of the need for enzymatic activation, nitroreductase expression is correlated with NIT susceptibility regardless of genetic background. Resistance against NIT evolves by chromosomal loss-of-function mutations in the nitroreductase genes [23,24]. Mutational deactivation of *nfsA* or *nfsB* alone only confers small resistance increases, i.e., ≤2× changes, but double mutants show high-level clinical resistances with 8× to 32× change of susceptibility [24]. Frequencies of resistance against NIT are low, and the antibiotic is increasingly used in clinical treatment [25,26].

At least 2 general mechanisms can lead to super-sensitivity against any given drug. The first mechanism is a relative increase of the active concentration of the drug at the target; the second is that the drug confers increased toxicity. These ultimate effects can each be achieved by a variety of upstream cellular processes. Higher active concentrations may result from altered uptake and efflux dynamics (increased net uptake) or faster drug activation. Increased toxicity can result from altered binding kinetics, or synergistic interactions with drug-response systems. The CS of a resistance mutation may be conferred by one or several co-occurring mechanisms.

This work shows that the CS to NIT that is observed in spontaneous mutants with resistance to tigecycline (*lon* mutation), mecillinam (*spoT* mutation), and protamine (*hemL* mutation) can be partly explained by their overexpression of the chromosomally encoded nitroreductases NfsA and NfsB. This common mechanism confers a moderate increase in susceptibility. Further susceptibility in the different individual mutants is a result of increased antibiotic uptake (*hemL* mutant) or an incompatibility of the resistance mutation with the cellular drug response (*lon* mutant). Our characterization of the genetic mechanisms of CS to a clinically relevant antibiotic supports the development of novel treatment approaches that work against resistant bacteria.

## Results

### Resistant mutants with CS against NIT

**Fig 1:**
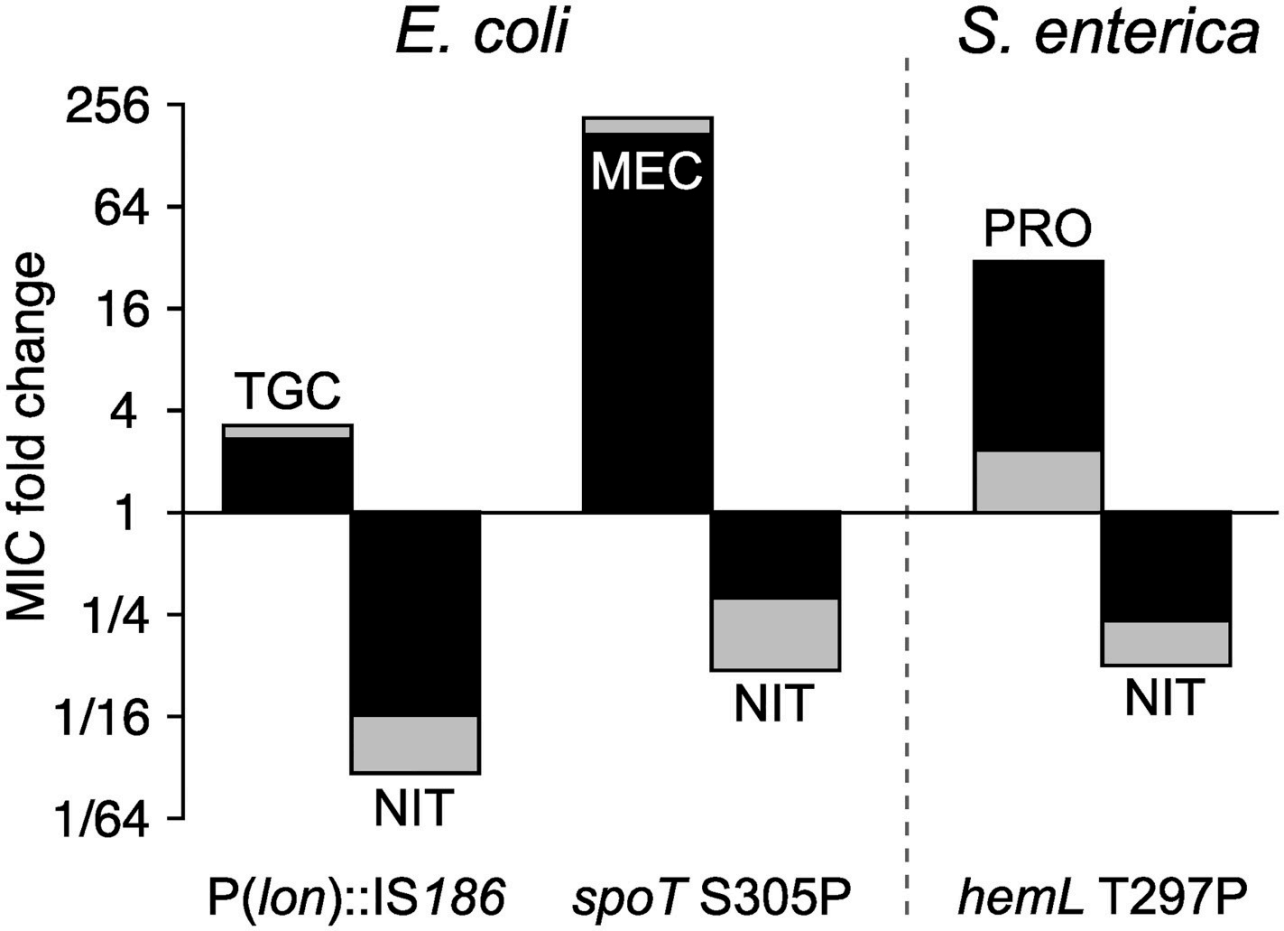
NIT CS. Three single-step mutants with resistance against 3 diverse antibiotics (upward bars) show CS to NIT (downward bars). Fold change of the MIC compared to susceptible wild-type strains. Light shading denotes an inoculum of 10^6^ cells; the overlaid dark shading corresponds to a larger inoculum of 10^8^ cells. Numerical data are available in S1 Data. CS, collateral sensitivity; MEC, mecillinam; MIC, minimum inhibitory concentration; NIT, nitrofurantoin; PRO, protamine sulfate; TGC, tigecycline.

As starting points for our mechanistic investigation, we assembled from our previous work a small but diverse set of 3 single-step mutants that were resistant to either tigecycline, mecillinam, or protamine but all showed CS to NIT (Fig 1). Susceptibility was measured as fold-decrease of the minimum inhibitory concentration (MIC) for the antibiotics compared to the isogenic parental strains. *E*. *coli* has an MIC of 4 mg/l, and the *S*. *enterica* wild type has an MIC of 12 mg/l. The *E*. *coli* wild type displays heteroresistance to NIT, i.e., that a small subpopulation of cells can grow and form colonies at 6–8 mg/l NIT. The MIC values reported in this paper characterize the main population.

Mutant 1 (DA19163) is an *E*. *coli* mutant with an insertion of an insertion sequence transposable element in the promoter region of the *lon* protease [16]. Mutant 1 was obtained by selection with tigecycline, which is a tetracycline derivate that inhibits protein translation and is used as a last-line drug for complicated infections. The *lon* mutant shows reduced susceptibility towards tigecycline but has a 16×-lower MIC to NIT (Fig 1). Mutant 2 (DA28438) is an *E*. *coli spoT* mutant that was obtained by selection with mecillinam (amdinocillin), towards which it has high-level mecillinam resistance and an associated CS to NIT with a 3× reduced MIC (Fig 1) [18]. Mecillinam is a β-lactam antibiotic that blocks cell wall synthesis, and it is widely used for the treatment of urinary tract infections in Scandinavia. This mutant has a greatly reduced growth rate and is genetically unstable, as is evident from the frequent appearance of larger-sized revertant colonies. Mutant 3 (DA10886) is an *S*. *enterica* small-colony variant with a single nucleotide substitution in *hemL* that was obtained by selection for resistance against the cationic antimicrobial peptide protamine sulfate and shows 4× CS to NIT (Fig 1) [17]. Protamine disrupts the integrity of the cytoplasmic membrane, leading to a collapse of membrane potential [27].

### CS caused by increased antibiotic uptake

**Fig 2:**
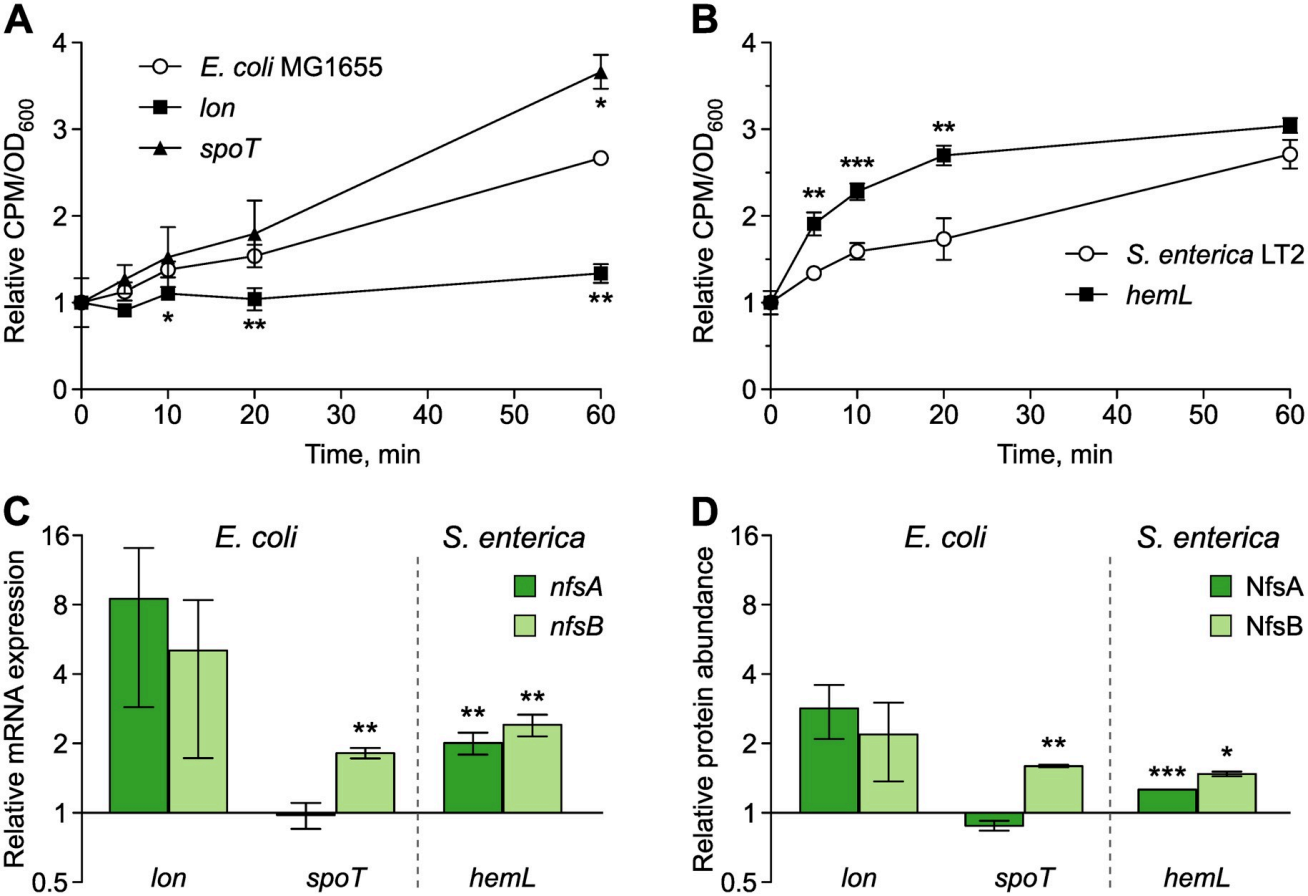
Mechanisms of CS to NIT. Uptake dynamics of radioactively labelled NIT measured as CPM in washed cell pellets of *E*. *coli* (A) and *S*. *enterica* (B). Measurements are normalized by the OD_600_ of the sample and expressed relative to the first measurement (*n* = 3 biological replicates). (C) mRNA expression of *nfsA* and *nfsB* relative to wild-type strains of *E*. *coli* and *S*. *enterica* measured with qRT-PCR (mean ± SD, *n* = 3 biological replicates). (D) Relative abundance of the proteins NfsA and NfsB measured with mass spectrometry (mean ± SD, *n* = 2 biological replicates). Student *t* test, ***P < 0.001, **P < 0.01, *P < 0.05. The numerical data for this figure and the full proteomic data sets are available in S1 Data. CPM, counts per minute; CS, collateral sensitivity; NIT, nitrofurantoin; OD_600_, optical density at 600 nm; qRT-PCR, quantitative reverse transcription PCR.

We first tested the hypothesis that the resistance mutations in *lon*, *spoT*, and *hemL* increased susceptibility to NIT through increased antibiotic uptake. We measured uptake of radioactive [^3^H]-NIT in the 3 mutants and the 2 parent strains (Fig 2A and 2B; see S1 Data for statistics). The uptake dynamics of [^3^H]-NIT was significantly accelerated in the *hemL* mutant compared to the *S*. *enterica* parent strain. The amount of intracellular [^3^H]-NIT rapidly increased in the *hemL* mutant, yielding significantly higher levels 5 min after addition of [^3^H]-NIT and approximately doubling after 20 min (Fig 2B). The uptake dynamics of the *spoT* mutant was not significantly different from that of the *E*. *coli* parent strain, with the exception of a small increase at the last measured time point (Fig 2A, S1 Data). The *lon* mutant showed significantly reduced [^3^H]-NIT uptake (Fig 2A, S1 Data). These results suggest that the CS of the *hemL* mutant is at least partly explained by increased uptake of the antibiotic but that other mechanisms account for CS in the *spoT* mutant, or may even have to compensate for the decreased NIT uptake in the *lon* mutant.

### CS caused by increased prodrug activation

We next tested whether the CS against NIT was due to increased prodrug activation through elevated expression of the nitroreductase enzymes NfsA and NfsB. Relative transcription of *nfsA* and *nfsB* in *E*. *coli*, and their homologs in *S*. *enterica* (*mdaA* and *nfnB*, hereafter called *nfsA* and *nfsB*), was measured using quantitative reverse transcription PCR (qRT-PCR). The results indicated increased transcript levels in the 3 mutants compared to their respective parent strains (Fig 2C), potentially indicating a common mechanism for CS against NIT in the 3 mutants of diverse evolutionary history. Both *nfsA* and *nfsB* showed significantly higher transcript levels in the *hemL* mutant (S1 Data). The *spoT* mutant had significantly elevated expression of *nfsB* only (S1 Data). The highest relative increase in transcript levels was obtained for the *lon* mutant, with a mean increase of 8× and 5× for *nfsA* and *nfsB*, respectively. The *lon* mutant showed high variation between biological replicates (*nfsA*: 4.4×–14.9×; *nfsB*: 2.6×–8.8×) such that the effect was not statistically significant, despite its likely stronger biological effect (S1 Data). To confirm the overexpression of the nitroreductases at the protein level, we conducted global proteomic analyses using mass spectrometry. Altogether 2,390 high-confidence proteins were consistently present in the *E*. *coli* samples and 2,665 in the *S*. *enterica* samples (the data sets are provided in S1 Data), including both nitroreductases on which we focused our further analysis. The relative protein abundances of nitroreductases in the CS mutants compared to their respective wild-type strains (Fig 2D) strongly correlated with the qRT-PCR data (Pearson’s correlation, *t* = 7.99; *df* = 4; *P* = 0.0013; *R*^*2*^ = 0.941). Thus, protein levels of the nitroreductases NfsB (in the *spoT* mutant) or NfsA and NfsB (in the *lon* and *hemL* mutants) were significantly increased in the CS mutants compared to the parent strains (S1 Data). These data suggest that overexpression of *nfsA* and *nfsB* contributes to CS in all 3 mutants.

**Fig 3:**
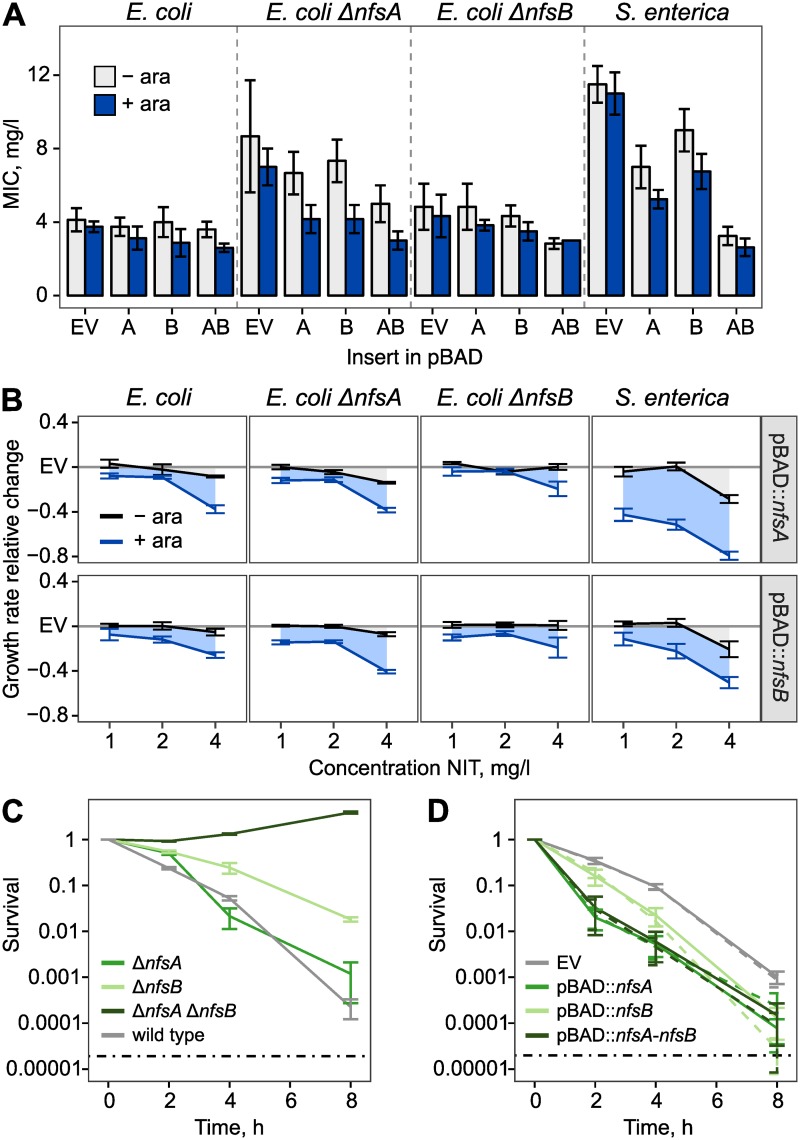
Nitroreductase expression increases susceptibility to NIT. (A) Susceptibility to NIT, as measured using Etest (bioMérieux, Marcy-l’Étoile, France) with a high inoculum of 10^8^ cells. The *nfsA* and *nfsB* open reading frames were cloned into the pBAD vector and expressed using induction by 0.2% arabinose (mean ± SD, *n* = 3–5 biological replicates). Single expression constructs are labelled “A” and “B” for *nfsA* and *nfsB*, respectively. Dual expression is labelled “AB.” (B) Expression of nitroreductases reduces exponential growth rate at low concentrations of NIT. Change of growth rate relative to EV (mean ± SEM, *n* = 3–5 biological replicates). (C) Deletion of nitroreductases promotes survival at high concentrations of NIT, here measured for *E*. *coli* using a time-kill experiment with 24 mg/l (mean ± SEM, *n* = 3 biological replicates). (D) Expression of nitroreductases reduces survival to NIT, here shown for the pBAD constructs in wild-type *E*. *coli* background and 24 mg/l NIT. Dashed lines indicate 0.2% arabinose, solid lines indicate absence of arabinose (mean ± SEM, *n* = 3 biological replicates). The grey dashed lines in (C) and (D) indicate the limit of detection, based on Poisson estimates. Numerical data are available in S1 Data. ara, arabinose; EV, empty vector; NIT, nitrofurantoin.

We next sought to determine the relative contribution of increased nitroreductase expression for CS. In particular, we tested whether increased expression of nitroreductases was sufficient to cause the very strong CS of the *lon* mutant. We made genetic constructs for single and dual overexpression of *nfsA* and *nfsB* (or their *S*. *enterica* homologs) from a medium-copy plasmid and measured the effect of nitroreductase expression on susceptibility to NIT. For single expression, the *nfsA* and *nfsB* open reading frames were individually cloned into the multiple-cloning site of the pBAD18 plasmid and placed under control of the arabinose-inducible promoter (P_BAD_). For dual overexpression, *nfsA* and *nfsB* were sequentially cloned into the multiple cloning site of pBAD18 to form a transcriptional fusion. We also constructed the *E*. *coli* strains Δ*nfsA*, Δ*nfsB*, and Δ*nfsA* Δ*nfsB* such that the chromosomal nitroreductases were deleted using a scar-free technique. The effects of nitroreductase expression for susceptibility to NIT were then determined using Etest (bioMérieux, Marcy-l’Étoile, France) measurements of MIC (Fig 3A), followed by several validation experiments that measured exponential growth rate at sublethal drug concentrations (1, 2, and 4 mg/l; Fig 3B) and time-kill dynamics using colony forming units (cfu) at 24 mg/l (Fig 3C and 3D). The main results of these analyses are as follows: (i) the expression of nitroreductases increases sensitivity to NIT in both species, and (ii) in wild-type backgrounds, the relative reductions of the MIC are limited to 1.4× in *E*. *coli* and 4.2× in *S*. *enterica* (effects are statistically significant, unpaired Wilcox rank sum test, empty vector versus *nfsA-nfsB* insert, + arabinose; for *E*. *coli*: W = 20, *P* = 0.01417; for *S*. *enterica*: W = 16, *P* = 0.02747). From these and our previous results, we conclude that increased nitroreductase expression is an important contributor to CS in the 3 mutants but that the relative effects are not sufficient to explain the full CS for the *spoT* mutant, and especially the *lon* mutant. A detailed description of the performed experiments and their results is provided in S1 Text.

### Regulators of nitroreductase expression

**Fig 4:**
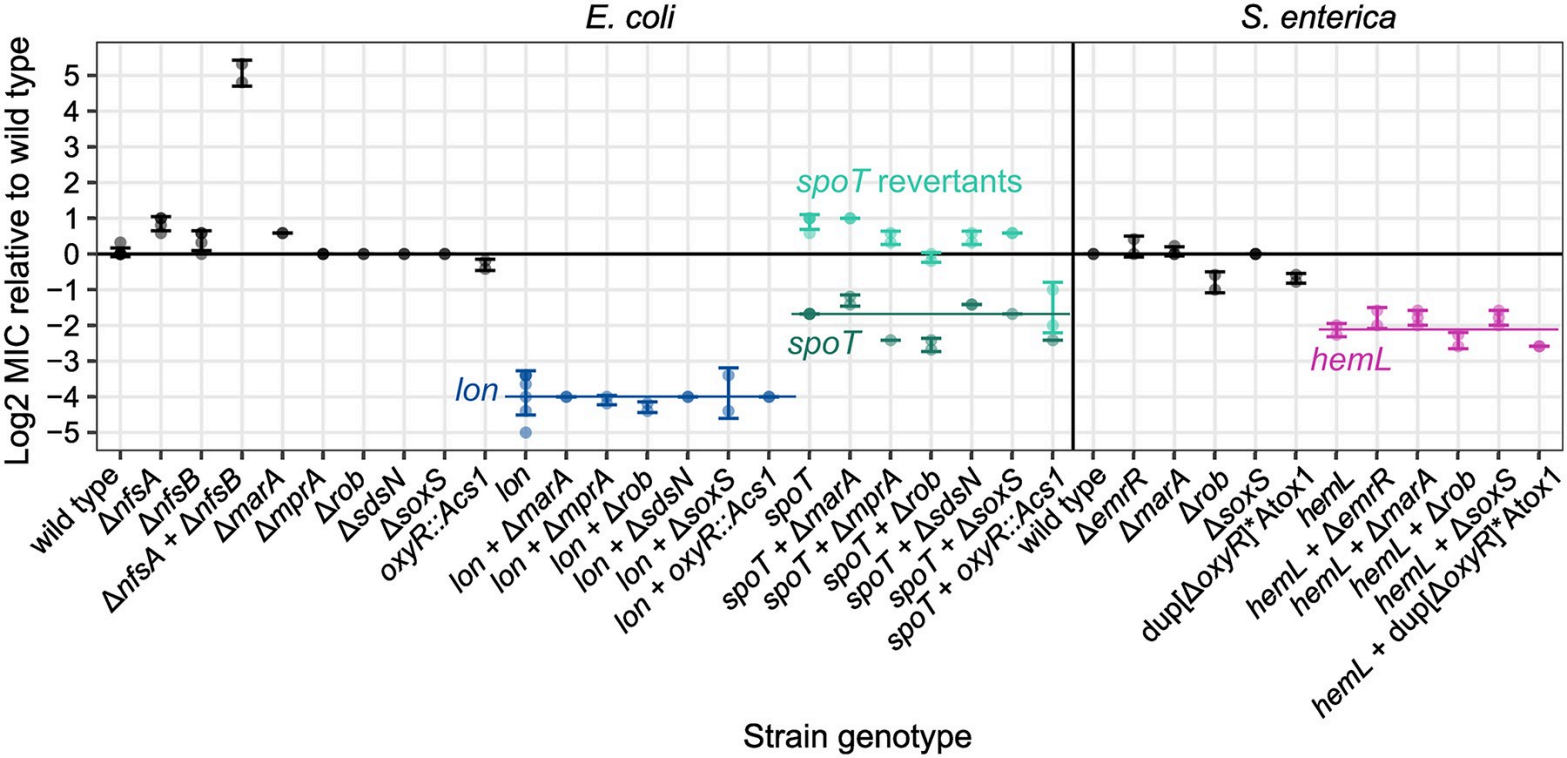
Regulators of CS. Change of susceptibility to NIT relative to wild-type strains for constructed single and double mutants. MIC was measured using Etest (bioMérieux, Marcy-l’Étoile, France) with a high inoculum of 10^8^ cells (mean ± SD, *n* ≥ 2 biological replicates). Coloured horizontal lines connect genotypes with the same CS mutation. The *spoT* mutant produced a high frequency of revertants, which are indicated in brighter colour and showed suppression of CS. “Acs1” and “Atox1” are resistance cassettes for genetic engineering. “dup” indicates a forced duplication. Numerical data are available in S1 Data. Acs1, amilCP-cat-sacB cassette 1; Atox1, amilCP-toxin cassette 1; CS, collateral sensitivity; MIC, minimum inhibitory concentration; NIT, nitrofurantoin.

Expression of *nfsA* and *nfsB* is highly regulated by several genes. In order to examine their importance for CS, we generated scar-free deletion mutants of all genes that are currently known to interact with *nfsA*, *nfsB*, or both (according to the *E*. *coli* database EcoCyc). Both *nfsA* and *nfsB* contain a *mar*-box that binds proteins of the *mar*-regulon (*marA*, *rob*, *soxS*). Transcription of *nfsA* is repressed by OxyR. It was recently demonstrated that expression of *nfsA* is also post-transcriptionally regulated by a small anti-sense RNA—*sdsN137*—in *E*. *coli* [28]. We further added the gene *mprA* (*emrR*) to our candidate list, because mutations in *mprA* were repeatedly selected in evolutionary experiments for high NIT resistance [29]. We transferred the scar-free deletions Δ*marA*, Δ*oxyR*, Δ*rob*, Δ*soxS*, Δ*mprA*, and Δ*sdsN137* into the respective mutant backgrounds and measured their MICs against NIT. Without exception, these measurements showed that CS was independent of the canonical regulatory pathways of *marA*, rob, *oxyR*, *soxS*, *mprA*, and *sdsN137* (Fig 4). Either deletion of potential regulatory genes did not affect the MIC of the CS mutants or the occurring minor changes were mirrored by the respective deletion mutants in the wild-type background (Fig 4). It is possible that the nitroreductase up-regulation is achieved by polygenic interactions, or by unknown regulators.

Altogether, expression of *nfsA* and *nfsB* increases susceptibility to NIT and thereby explains part of the CS effect in all 3 mutants. In *S*. *enterica*, the effect of nitroreductase expression for MIC was substantial (up to 4.2×) and may be sufficient to explain the remainder of the *hemL* sensitivity. The effects of increased nitroreductase expression were lower in *E*. *coli* (less than 2×), which strongly suggests that additional mechanisms contribute to the high sensitivity of the *spoT* mutant and the extraordinary sensitivity of the *lon* mutant.

### CS resulting from incompatibility with cellular drug response

**Fig 5:**
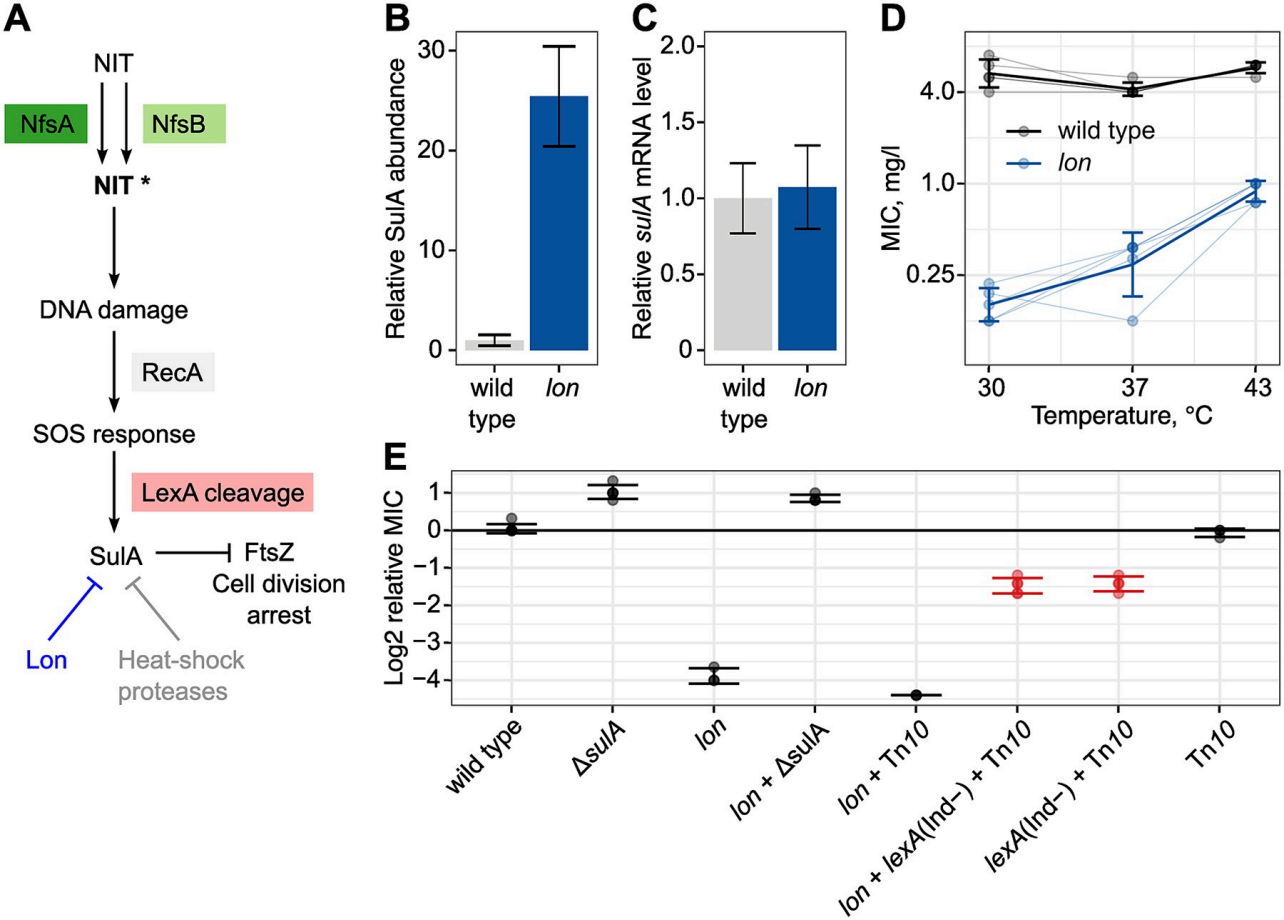
CS due to interference with the cellular drug response. (A) Model for elevated susceptibility to NIT in a *lon* mutant due to induction of the SOS response. (B) Relative accumulation of SulA protein in the *E*. *coli lon* mutant in drug-free medium (mean ± SD, *n* = 2 biological replicates). (C) Transcription of *sulA* is unchanged in the *lon* mutant in drug-free medium (mean ± SD, *n* = 3 biological replicates). (D) Partial suppression of CS at high temperature (mean ± SD, *n* = 5 biological replicates). (E) Relative change of MIC compared to wild-type *E*. *coli* in mutants of the SOS response. CS is completely suppressed by deletion of *sulA* and partially suppressed by a noninducible allele of *lexA* (mean ± SD, *n* = 3–5 biological replicates). Numerical data are available in S1 Data. CS, collateral sensitivity; MIC, minimum inhibitory concentration; NIT, nitrofurantoin; SulA, Suppressor of Lon.

The proteomics data of the *lon* mutant showed extensive changes in protein abundance. The strongest effect was on the abundance of Suppressor of Lon (SulA), which was increased 25× (Fig 5B), while the transcription of *sulA* was unchanged in the *lon* mutant (Fig 5C). SulA is a cell-division inhibitor involved in the SOS response. Under physiological conditions, dimeric LexA repressor proteins prevent expression of several genes, including *sulA*, that together compose the SOS response (for a review of the SOS response, see [30]). The occurrence of DNA damage is recognized by RecA protein, which then stimulates an autocleavage of LexA, resulting in a de-repression of the SOS function [30]. Variation in the LexA-binding affinities between operator sequences generates a sequential induction of different functions [30]. Induction of SulA expression is part of the late SOS response [30], and increased SulA levels stop replication and thereby allow the cell to perform DNA lesion repair. SulA is degraded by the Lon protease [31], and cell growth eventually resumes. Alternative proteases for SulA, such as ClpYQ, are induced by heat shock [32,33]. It has been demonstrated that NIT causes DNA damage and induces the canonical SOS response in *E*. *coli* MG1655 [29,34]. Based on this knowledge, we hypothesized that induction of the SOS response by NIT may contribute to the CS of the *lon* mutant. Upon induction of SOS, formed SulA would accumulate and not be degraded in a *lon* mutant, thereby blocking cell division and contributing to antibiotic toxicity (Fig 5A).

As a first test of this hypothesis, we characterized the temperature dependence of the CS of the *lon* mutant. The *E*. *coli* heat shock response triggers the expression of alternative proteases such as ClpYQ that can degrade accumulated SulA [32,33], potentially resulting in a suppression of CS. We measured susceptibility of the *lon* mutant at 30 °C, 37 °C, and 43 °C. The MIC for NIT showed strong temperature dependence, with lower susceptibility at high temperature (Fig 5D). The MIC at 43 °C was 0.75–1.0, which was 6× to 8× higher than the MIC of 0.125 at 30 °C. The *E*. *coli* wild-type MIC showed no temperature dependence (Fig 5D). We then tested whether CS was dependent on SulA by generating the scar-free deletion Δ*sulA* in *E*. *coli* and transferring the mutation to the *lon* mutant. The CS phenotype was suppressed in the resulting *lon*+Δ*sulA* double mutant (Fig 5E), supporting our model that the high CS in the *lon* mutant is caused by the accumulation of SulA following SOS induction. To confirm that CS was dependent on induction of the SOS response, we made use of a previously characterized, noninducible *lexA* allele (LexA1 Ind^−^), which we transferred to the *lon* strain. In this double mutant, a substantial amount of the CS was restored, i.e., the MIC was increased to 1.5 mg/l (6× increase), which was still 3× below the wild-type *E*. *coli* strain (Fig 5E). We conclude that the extraordinarily high CS of the *lon* mutant is explained by the activity of the SOS response. The high effect size of CS in this case is a consequence of increased NIT toxicity that is caused by a *sulA*-dependent interference of the cell’s own drug-response module with growth.

**Fig 6:**
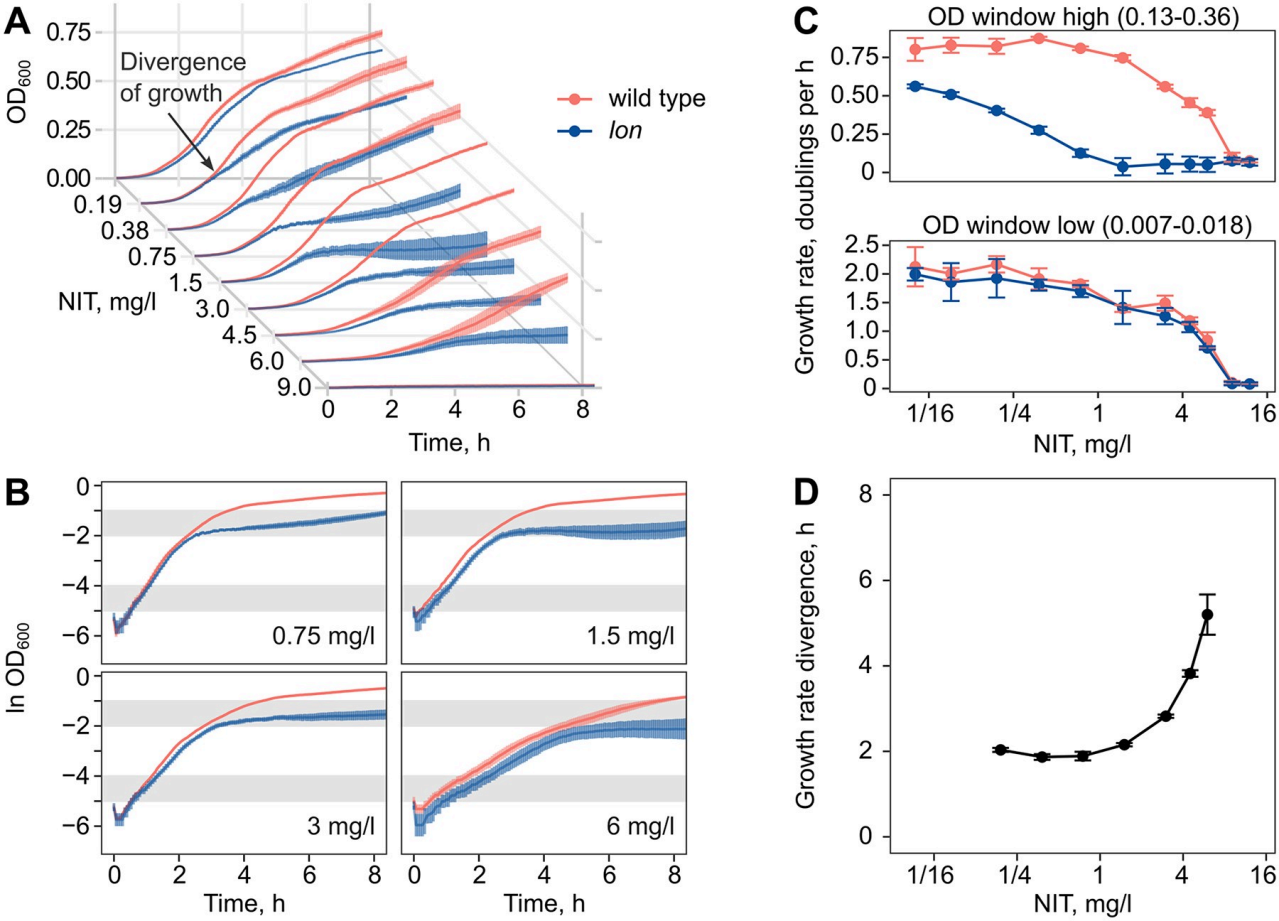
Temporal dynamics of CS in the *lon* mutant. Delayed establishment of inhibition at low concentrations of NIT inhibition in the *lon* mutant. (A) Growth kinetics of the *E*. *coli* wild type and the *lon* mutant diverge after several hours of equal growth (mean ± SEM, *n* = 3 biological replicates). (B) Growth kinetics with log-transformed OD_600_ data for a subset of concentrations. The shaded grey regions indicate the OD_600_ windows that were used for the determination of exponential growth rate. (C) Dose-dependent inhibition of exponential growth rate in early (bottom) and late (top) OD_600_ windows (mean ± SD, *n* = 3 biological replicates). (D) Time point of exponential growth rate divergence between the 2 strains (mean ± SD, *n* = 3 biological replicates). Numerical data are available in S1 Data. CS, collateral sensitivity; NIT, nitrofurantoin; OD_600_, optical density at 600 nm.

Through this mechanism, strong growth inhibition in the *lon* mutant should establish after a certain “gap time” of comparatively uninhibited growth, since *sulA* expression is induced in the late stage of the SOS response [30]. To test this prediction, we measured the growth inhibition dynamics upon addition of subinhibitory concentrations of NIT. Growth dynamics precisely matched that of the *E*. *coli* wild type for 2–5 h and then diverged as a result of inhibition build-up (Fig 6A), in agreement with our model. An analysis of the dynamics with log-transformed data indicated a pronounced reduction of growth rate at late exponential phase in the *lon* mutant (Fig 6B). To quantify the dynamics, we calculated dose-response curves for early growth during the gap time and, thereafter, the phase of delayed inhibition (Fig 6C). The dose-response curves of wild type and *lon* mutant were overlapping for the early phase but clearly separated in the late phase, such that equivalent growth rates of the *lon* mutant were shifted to 6× lower concentrations. We quantified the precise time point of growth rate divergence between the 2 strains, using a sliding window approach. This analysis confirmed the onset of inhibition after 2–5 h of uninhibited growth in the *lon* mutant (Fig 6D). In contrast, the *hemL* and *spoT* mutants showed immediate inhibition (S1 and S2 Figs), which for the *hemL* mutant is consistent with the CS mechanism of increased drug uptake.

## Discussion

Starting with 3 very different resistant mutants that showed CS to NIT enabled us to develop a mechanistic understanding of NIT hypersensitivity. The 3 mutants from 2 species (*E*. *coli* and *S*. *enterica*) differed in their resistance characteristics (β-lactam, tigecycline, antimicrobial peptide). Using a functional genetics approach, we determined a number of shared and individual molecular mechanisms of CS to NIT for the 3 drugs. These mechanisms can explain the observed high degrees of hypersensitivity fully for 2 mutants (*hemL* and *lon*) and partially for the third mutant (*spoT*). The unexplained portion of CS in the *spoT* mutant could be resolved by further work, but this would not change the main result of this paper, which is that CS is the result of multiple mechanisms.

We observed a transcriptional up-regulation of nitroreductase activity in all 3 mutants such that the oxygen-insensitive nitroreductases NfsA and/or NfsB were overexpressed at both the transcript and protein level (Fig 2C and 2D). With deletion strains and overexpression constructs, we were able to show that this activity increases susceptibility to NIT roughly 1.4× (*E*. *coli*) to 4.2× (*S*. *enterica*) as measured by MIC. These effect sizes should be seen as upper limits because the achieved expression levels from the pBAD plasmid are likely larger than those that occurred in the CS mutants. Loss-of-function mutations in nitroreductase genes are the primary resistance mechanisms against NIT [24]. The relationship of nitroreductase expression and NIT susceptibility is clearly established [35], but the relative importance of the expression of either gene is less understood. Here, we resolve some of the redundancy. NfsB has the larger effect on killing rate, but NfsA is critical for initial survival (Fig 3C and 3D). This time-kill dynamics is consistent with the observed mutational trajectory of *nfsA* mutations appearing before *nfsB* mutations [24]. We went through a great effort to identify the genetic regulators for the overexpression of nitroreductases, producing scar-free deletions of all known interactors in the 2 wild-type strains and the 3 CS mutants. However, we could not explain the up-regulation by the activity of any single gene. Thus, increased nitroreductase expression may either result from the activity of an unknown regulator or a more complex polygenic interaction. While an investigation with double, triple, and quadruple mutants is generally possible—and potentially interesting with respect to the *mar*-regulon—it is beyond the scope of this manuscript. Moreover, nitroreductase overexpression only partly accounts for the CS to NIT.

The *hemL* mutant showed significantly increased antibiotic uptake rates (Fig 2B). Antibiotic uptake is the focus of previously characterized systems. In the best studied example of CS, β-lactam hypersensitivity in aminoglycoside-resistant *trkH* mutants is explained by the reduced activity of the AcrAB-TolC multidrug efflux pump as a consequence of a reduced membrane potential [8]. These changes likely increased the intracellular accumulation of β-lactams that are substrates of the pump, although the accumulation was not directly measured in that study. Here, we conducted direct measurements of the uptake dynamics using radiolabelled NIT and could thereby further support this previous model. There are indications that an altered ratio of drug uptake and efflux also explains the CS of ciprofloxacin-resistant *nfxB* mutants in *Pseudomonas aeruginosa* [36,37]. Uptake dynamics that increase the active intracellular concentration of an antibiotic appear to be a common mechanism of CS.

The highest CS in our study occurred in the *lon* mutant and could be explained by particularly high nitroreductase levels and an intriguing interference of the native drug-response system with growth. NIT causes DNA damage to which the cell responds by mounting the SOS response system. As part of the SOS activity, SulA accumulates and stops growth. At physiological temperatures, growth resumption depends on *lon*-mediated SulA degradation. High stability of SulA protein in *lon* mutants explains at least a 6× decrease in MIC. Our results are consistent with the previous findings that *lon* mutations are lethal in SOS-constitutive strains with null mutations in *uvrD* [38] and that *sulA*^+^ multicopy plasmids cannot be established in *lon* mutants [39]. The *lon* mutation caused a 16× to 35× decrease in MIC, indicating a high potential for therapy.

Drug switches can restore control of evolving pathogen populations and may be preplanned according to CS characteristics. We here investigated the possibility of switching to NIT. NIT is a good last drug in a switching regime because the CS effects are unusually strong (i.e., 3× to 35× decreases in MIC) and occur in phenotypically diverse resistant strains. Furthermore, NIT shows high stability to resistance evolution [29] and low resistance levels in the population [26]. Altogether, drug switches to NIT seem as a promising treatment possibility that is supported by the common mechanism of increased nitroreductase expression. Yet we also found that the most effective treatment effects were due to specific co-occurring mechanisms. We anticipate that the here-provided mechanistic basis for CS will stimulate the development of efficient drug switches to combat resistance evolution.

## Methods

### Strains and media

Work was conducted using wild-type strains of *E*. *coli* K-12 MG1655 (DA5438) and *S*. *enterica* serovar Typhimurium LT2 (DA6192). The single-step CS mutants DA19163 (*E*. *coli*, *P*_*lon*_::IS*186*), DA28438 (*E*. *coli*, *spoT* S305P), and DA10886 (*S*. *enterica*, *hemL* T297P) were derived from these parent strains, as previously described [16–18]. All incubations were performed with orbital shaking (190 rpm) at 37 °C, unless otherwise specified. Cultures were grown in lysogeny broth with 10 g/l NaCl (LB; Sigma, Refs. L3522-1KG, 56062–2.5KG) for standard growth and in Müller-Hinton media (Becton Dickinson, Sparks, MD; Refs. 275730, 225250) for antibiotic susceptibility testing. Measurements of exponential growth rate and killing rate were conducted in LB media lacking yeast extract (tryptone broth [TB]; Oxoid, Hants, UK; Ref. LP0042) because the P_BAD_ promoter is active during the early exponential growth phase in TB but not in LB. For selective media, antibiotics were added at the following concentrations: ampicillin 100 mg/l, chloramphenicol 12.5 or 30 mg/l, tetracycline 10 mg/l, and trimethoprim 10 mg/l. Antibiotic stock solutions were prepared according to manufacturer instructions, sterile filtered, and stored in small aliquots for single use at −20 °C. NIT (Sigma-Aldrich, Ref. N7878-10G) stocks were prepared in DMSO at 10 mg/ml.

### MIC determination

MICs were determined using Etest strips (bioMérieux, Marcy-l’Étoile, France), or Liofilchem MIC test strips (liofilchem srl, Roseto degli Abruzzi, Italy). Cultures were prepared from single colonies in Müller-Hinton broth and overnight incubation. The cultures were then diluted in 0.9% saline 1:20 for a standard inoculum density of 10^8^ cfu/ml (equivalent to 0.5 McFarland), or 1:1000 for a lower inoculum density of approximately 10^6^ cfu/ml. Heteroresistance is only detectable at the higher inoculum density. The prepared cell suspensions were streaked onto Müller-Hinton agar using a sterile cotton swab. Etest strips were applied, and the plates were incubated for 20 h, after which the MIC was read according to manufacturer instructions. For protamine sulfate, MICs were determined in glass vials using broth microdilution in ISO-Sensitest broth (Oxoid, Hants, UK; Ref. CM0473) supplemented with 0.2% glucose, as previously described [17].

### Radiolabelled NIT uptake assay

Overnight cultures were diluted 1:100 in LB and grown to an optical density at 600 nm (OD_600_) of 0.3 to 0.4 and then supplemented with [^3^H]-NIT (Larodan, Solna, Sweden) to a final activity of 100 nCi/ml. After 0, 5, 10, 20, and 60 min, 1-ml aliquots of the cultures were transferred to Eppendorf tubes and pelleted by centrifugation (13,500*g*, 3 min, room temperature), and the supernatant was decanted. The pellets were washed twice with phosphate buffered saline (PBS), then resuspended in 0.5 ml PBS and transferred to 1 ml Optiphase HiSafe 3 scintillation cocktail (PerkinElmer, Waltham, MA). The samples were measured with a Tri-Carb 2810 TR liquid scintillation analyser (PerkinElmer) over the duration of 1 min per sample. The counts were normalized to OD_600_ values determined after the 0-, 5-, 10-, 20-, and 60-min incubation with [^3^H]-NIT to correct for differences in cell density. The normalized counts were expressed relative to time point 0 min.

### qRT-PCR

Overnight cultures were diluted 1:100 in 30 ml LB and incubated until OD_600_ ≈ 0.3. One millilitre of the culture was added to 2 ml RNAprotect Bacteria Reagent (Qiagen, Hilden, Germany; Ref. 76506). Total RNA was extracted using the RNeasy Mini Kit (Qiagen, Hilden, Germany; Ref. 74104). Chromosomal DNA was removed using the DNase Turbo DNA-free kit (Invitrogen, Thermo Fisher, Vilnius, Lithuania; Ref. AM1907). After the DNase treatment, the total RNA extract was subjected to gel electrophoresis for quality control. The amount of 500 ng of DNA-free RNA was reverse transcribed using the High Capacity Reverse Transcription kit (Applied Biosystems, Thermo Fisher, Vilnius, Lithuania; Ref. 4368814) in a total reaction volume of 20 μl. Relative cDNA levels were measured using PerfeCTa SYBR Green SuperMix (Quanta Biosciences, Beverly, MA; Ref. 95072–012) in an Eco Real-Time PCR System (Illumina, San Diego, CA; Ref. 1010180). Each primer pair (see S1 Table for nucleotide sequences) was tested for efficiency in a set of six 10× dilutions. The genes *hcaT* and *cysG* were used as reference genes in the calculations for relative expression. Expression level was calculated as follows: 2^(geometric mean of Cq values for the reference genes − Cq of the target gene). For relative expression, expression level of the CS mutant was normalized by the expression level of the wild type. All measurements were repeated for 3 biological and 3 technical replicates in the form of 10^−1^, 10^−2^, and 10^−3^ dilutions of the template.

### Nitroreductase overexpression

The open reading frames of *nfsA* and *nfsB* were individually amplified from the *E*. *coli* MG1655 chromosome with primers containing restriction sites for EcoRI (gaattc) in the forward primer together with the ribosome binding site AGGAGG and a 6-nt spacer TAAATA) and XbaI (tctaga) in the reverse primer. Purified PCR products were digested and cloned into the pBAD18 vector (GenBank accession: X81838, ampicillin resistance, expression from the P_BAD_ promoter), digested with the same enzymes. The transcriptional fusion *nfsA-nfsB* was generated by cloning the *nfsB* open reading frame (together with the added ribosome binding site and spacer) into the pBAD::*nfsA* vector, following a second digestion with XbaI (tctaga) and HindIII (aagctt). The constructs were verified by Sanger sequencing and transformed into the target strains by electroporation using a Gene Pulser Xcell (BIO-RAD) at 1.8 kV, 25 μF, 200 Ω, with a 1-mm cuvette. The same procedures were applied for the *S*. *enterica* serovar Typhimurium LT2 homologs *nfnB* and *mdaA*, yielding single- and double-overexpression constructs in pBAD for this species.

### Strain list

See S2 Table for an overview of all used strains, as well as the strains constructed in this study. The nucleotide sequences of the DNA oligos used for the constructions are provided in S3 Table.

### Sanger sequencing

Manipulated chromosomal regions were PCR amplified (DreamTaq, Thermo Fisher, Ref. K1082). The PCR products were purified using the GeneJet Gel Extraction Kit (Thermo Fisher, Ref. K0692) and sent for Sanger sequencing using the Mix2Seq service (Eurofins Genomics, Ebersberg, Germany).

### Interaction partners of *nfsA* and *nfsB*

To study the regulation of *nfsA* and *nfsB* overexpression in the CS mutants, we constructed knock-out deletion strains of all known regulators of *nfsA* and *nfsB*. A list of all genes known to interact with *nfsA* and/or *nfsB* was obtained from EcoCyc version 23.0 (https://ecocyc.org) [40] on June 17, 2019. Known regulators of *nfsA* are MarA, Rob, SoxS, OxyR, and SdsN137; the known regulators of *nfsB* are MarA, SoxS, and Rob. To this list we added MprA and its homolog EmrR in *Salmonella* because of their recent implications in the resistance evolution to NIT [29].

### Strain construction

Scar-free chromosomal gene deletions were constructed by duplication-insertion recombineering [41] and transduction with P1 phage for *E*. *coli* and P22 for *S*. *enterica*. Altogether, the process consists of 3 sequential lambda red recombinations, followed by the transduction of the finished chromosomal construct to the target recipient strains, and selectable spontaneous scar-free excision of the marker. In brief, DNA oligonucleotides are designed to contain primer binding sites to amplify a resistance cassette (p1: GTGTAGGCTGGAGCTGCTTC; p2: CATATGAATATCCTCCTTAGTTCC) and overhangs with homology to the chromosome. Recombination between PCR products (Phusion Green High-Fidelity DNA Polymerase, Thermo Fisher, Ref. F534L) and the chromosome enables allele swaps, whereby genes can be deleted with single-nucleotide precision. The recombination is catalysed by the temperature-inducible expression of the lambda red system that is provided on a plasmid. The chromosomal resistance marker is then replaced in a second recombination, against a single-strand DNA oligo that is designed to leave no scar in the genome. The finished construct is then duplicated in the chromosome by help of “forced-duplication oligonucleotides,” again through lambda red recombination. The duplication is transferred to the target strain (wild type, CS mutant, or other mutant) using transduction. The duplication spontaneously excises from genome due to the instability of inverted repeats, and excised variants can be selected. The constructions were generally performed with the “Acatsac1” cassette (GenBank MF124798) that contains chloramphenicol resistance and the blue chromoprotein *amilCP* from *Acropora millepora* for positive selection and the *sacB* gene from *Bacillus subtilis* for negative selection (lethal on agar plates with 2% sucrose). Selection with “Acatsac1” did not work in the *S*. *enterica hemL* mutant, but we were successful with the “Atox1” cassette (GenBank accession MN207489), which uses trimethoprim resistance and a rhamnose-inducible toxin (lethal at 0.2% L-rhamnose) for positive and negative selection, respectively. Transformations were performed by electroporation, using a Gene Pulser Xcell (BIO-RAD) at 2.5 kV, 25 μF, 200 Ω, with a 1-mm cuvette, and transformants were allowed to recover in SOC medium (tryptone 20 g/l, yeast extract 5 g/l, NaCl 0.58 g/l, KCl 0.186 g/l, 2 mM glucose, 2 mM MgSO_4_) for 3 h at 30 °C. The methodology was applied to construct a total of 33 final strains, as described in the strain list (S2 Table). The finished deletion constructs were verified by Sanger sequencing. No unwanted mutations were detected.

### Strain construction design considerations

The strains were constructed so as not to disturb the expression of off-target genes in the chromosomal neighbourhood. For overlapping open reading frames, we designed the recombineering oligonucleotides to leave behind the overlapping region in reading frame. If promoter regions for other genes—as identified by RegulonDB version 10.5 (http://regulondb.ccg.unam.mx) [42]—were located within the gene to be deleted, we left those parts undisturbed, in frame, and with their native start and stop codons. More information is provided in the strain list (S2 Table). For *oxyR*, scar-free deletions could not be generated although the gene could be exchanged for a resistance cassette.

### Transfer of *lexA1* mutation to generate SOS noninducible strains

A noninducible allele of *lexA* (LexA1 Ind^−^) was transferred to the *E*. *coli* MG1655 wild type (DA5438) and the *E*. *coli lon* mutant (DA19163) by P1 transduction and selection for a chromosomal tetracycline resistance marker (*malF3089*::Tn*10*; 12.5 mg/l tetracycline), using the donor strain SK1382 that originates from the Abram Aertsen laboratory at KU Leuven, Belgium [43]. Transductants were pure streaked and screened by Sanger sequencing for transfer of the correct *lexA1* allele. As controls, we transduced the tetracycline resistance marker into the wild-type and *lon* mutant strains with the inducible LexA.

### Growth rate measurements

Overnight cultures for 3–5 biological replicates were prepared from single colonies in TB (for work with pBAD, Fig 3) or Müller-Hinton broth (Fig 6). For pBAD construct experiments, the medium was supplemented with 100 mg/l ampicillin for plasmid maintenance and 0.2% L-arabinose to induce expression of the P_BAD_ promoter, if necessary. Assay conditions (antibiotics, arabinose) were prepared in large volume, of which 1-ml portions were aliquoted into Eppendorf tubes that were inoculated with 1-μl of dense bacterial culture (1:1000). The complete assay mixture was then vortexed and dispensed into a Bioscreen honeycomb plate wells (total assay volume of 300 μl), generating 2–3 technical replicates for each assay condition. Honeycomb plates were transferred to a Bioscreen C plate reader (OY Growth Curves, Helsinki, Finland; Ref. FP-1100-C) for incubation (37 °C, orbital shaking with medium amplitude and normal speed) and measurements of OD_600_ every 4 min for a total of 24 h. Data were analysed using R [44]. In brief, background values were subtracted from raw OD_600_ reads using wells with uninoculated medium. Then, the background-corrected values were log transformed (ln_OD_600_), and exponential growth rates (doublings per hour) were calculated in an OD_600_ window of fully exponential growth, typically in the OD_600_ range of 0.0067 (ln −5) to 0.0183 (ln −4). To verify exponential growth, we calculated the goodness of fit *R*^*2*^ for ln_OD_600_ to time. For the work in Fig 3, a total of 646 growth curves were analysed for which *R*^*2*^ ranged from 0.934 to 0.998 with a mean of 0.987. For the analysis in Fig 3B, the growth rates of strains with overexpression constructs were normalized by the corresponding mean growth rate of the empty vector controls. The values reported in Fig 3B are the change of relative growth rate, expressed as relative growth rate − 1.

### Time of growth rate divergence

The time point of growth divergence between wild type and CS mutant was quantified using a sliding window approach. Growth rates were calculated using log-transformed OD_600_ values (ln_OD_600_) in a sliding window across time (windows size of 1 h, or 15 measurements). The absolute difference in growth rates in divisions per hour was then measured by subtracting the window average mutant growth rate from the window average wild-type growth rate. Growth rates were considered as diverged if the difference exceeded a threshold of 0.25 divisions per hour, which corresponds to the substantial reduction equivalent to 10% of wild-type maximum exponential growth rate in drug-free conditions. The time point of growth rate divergence as reported in Fig 4D is the temporally first instance of growth rate divergence above the threshold. The reported times correspond to the lower bound of the sliding window.

### Time kill

Overnight cultures for 3 biological replicates were prepared from single colonies in TB, supplemented with 100 mg/l ampicillin added for plasmid maintenance, if necessary. Assay conditions (NIT at 24 mg/l corresponding to 6× MIC, induction with 0.2% L-arabinose) were prepared in large volume, and 1 ml of mixture was aliquoted into 10-ml culture tubes and inoculated with dense overnight culture to achieve a starting density of 3 × 10^6^ cfu/ml (1:200 dilution). Tubes were incubated in a shaking water bath (37 °C, 184 rpm orbital shaking). Samples were collected after 0, 2, 4, and 8 h, serially diluted in 0.9% saline and plated onto LB agar with sterile glass beads (4 mm, 7 beads per plate). cfu counts were determined after 20–24 h of incubation at 37 °C. Survival was expressed as cfu/ml of a sample relative to its starting density.

### Sample preparation for global proteomic analysis

Overnight cultures of 2 biological replicates were diluted 1:100 in 30 ml of prewarmed LB and incubated until OD_600_ ≈ 0.3. Twenty millilitres of the culture was pelleted by centrifugation (5,000*g*, 4 °C, 10 min), washed twice with PBS, and frozen at −80 °C for further sample preparation.

### Protein extraction for proteomic analysis

Cell pellets were homogenized on a FastPrep-24 instrument using the lysis matrix B (MP Biomedicals, Solon, OH) for 4 repeated 40-s cycles at 6.5 m/s in 200 μl of the buffer containing 2% sodium dodecyl sulfate and 50 mM triethylammonium bicarbonate (TEAB). Samples were centrifuged at 6,200*g* for 20 min. The supernatants were transferred to clean tubes. The lysis tubes were washed with 110 μl of the lysis buffer and centrifuged again. The supernatants were combined with the corresponding lysates from the previous step. Protein concentration in the combined lysates was determined using Pierce BCA Protein Assay Kit (Thermo Scientific, Ref. 23225) and the Benchmark Plus microplate reader (BIO-RAD) with bovine serum albumin solutions as standards.

### Tryptic digestion and tandem mass tag labelling

Aliquots containing 50 μg of total protein were taken from each sample and incubated at 60 °C for 30 min in the lysis buffer with DL-dithiothreitol at 100 mM final concentration. The reduced samples were processed using the modified filter-aided sample preparation method [45]. In short, reduced samples were diluted to 500 μl by addition of 8 M urea, transferred onto Nanosep 10k Omega filters (Pall Life Sciences, Ann Arbor, MI; Ref. OD010C33) and washed 2 times with 200 μl of 8 M urea. Alkylation of the cysteine residues was performed using 10 mM methyl methanethiosulfonate diluted in digestion buffer (1% sodium deoxycholate, 50 mM TEAB) for 30 min at room temperature, and the filters were then repeatedly washed with digestion buffer. Trypsin (Pierce Trypsin Protease, MS Grade, Thermo Scientific, Ref. 90059) in digestion buffer was added at the trypsin:protein mass ratio of 1:100, and the samples were incubated at 37 °C for 3 h; another portion of trypsin (1:100) was added and incubated overnight. The peptides were collected by centrifugation and labelled using Tandem Mass Tag (TMT) reagents (Thermo Scientific). The labelled samples were combined into 2 separate pooled samples for the *E*. *coli* and *S*. *enterica*; the pooled samples were concentrated using vacuum centrifugation, and sodium deoxycholate was removed by acidification with 10% trifluoroacetic acid and centrifugation. The TMT-labelled samples were fractionated using Pierce High pH Reversed-Phase Peptide Fractionation Kit (Thermo Scientific, Ref. 84868) according to the manufacturer’s protocol. Eight fractions were collected using the elution solvents consisting of 0.1% of aqueous trimethylamine solution and 7%, 10%, 13%, 16%, 19%, 2%, 25%, and 50% of acetonitrile. The fractions were dried on Speedvac and reconstituted in 15 μl of 3% acetonitrile, 0.2% formic acid for LC-MS/MS analysis.

### LC-MS/MS analysis

The fractions were analysed on an Orbitrap Fusion Tribrid mass spectrometer interfaced with an Easy-nLC1200 liquid chromatography system (both Thermo Fisher Scientific). Peptides were trapped on an Acclaim Pepmap 100 C18 trap column (100 μm × 2 cm, particle size 5 μm, Thermo Fischer Scientific) and separated on an in-house packed analytical column (75 μm × 30 cm, particle size 3 μm, Reprosil-Pur C18, Dr. Maisch GmbH, Ammerbuch, Germany) using a linear gradient from 5% to 35% B over 75 min followed by an increase to 100% B for 5 min, and 100% B for 10 min at a flow of 300 nl/min. Solvent A was 0.2% formic acid in water, and solvent B was 80% acetonitrile and 0.2% formic acid. MS^1^ scans were performed at 120,000 resolution and m/z range of 380–1,380. The most abundant doubly or multiply charged precursors were selected with a “top speed” cycle of 3 s—fragmented by collision-induced dissociation at 35 collision energy with a maximum injection time of 50 ms—and were detected in the ion trap followed by multinotch isolation of the top 7 MS^2^ fragment ions within the *m/z* range of 400–1,400, MS^3^ fragmentation by higher-energy collision dissociation (HCD) at 65% collision energy, and detection in the Orbitrap at 50,000 resolution. Precursors were isolated in the quadrupole with a 0.7 m/z isolation window and dynamic exclusion within 10 ppm for 45 s.

### Proteomic data analysis

Identification and relative quantification of protein abundance was performed using Proteome Discoverer version 2.2 (Thermo Fisher Scientific). The *E*. *coli* MG1655 and *S*. *enterica* serovar Typhimurium LT2 databases were downloaded from Uniprot (December 2017) and supplemented with the common proteomic contaminants. A database search was performed using the Mascot search engine version 2.5.1 (Matrix Science, London, UK) with MS peptide tolerance of 10 ppm and fragment ion tolerance of 0.6 Da. Tryptic peptides were accepted with 1 missed cleavage; methionine oxidation was set as a variable modification; and cysteine methylthiolation, TMT-6 on lysine, and peptide N-termini were set as fixed modifications. Percolator was used for peptide-spectrum match validation with the false discovery rate threshold of 1%. TMT reporter ions were identified in the MS^3^ HCD spectra with 3 mmu mass tolerance, and the TMT reporter intensity values for each sample were normalized on the total peptide amount. Only the unique identified peptides were considered for the relative quantification, which was our sole analysis. We did not perform a systems-level investigation of the mutant proteomes, but this should be possible with the data sets provided in S1 Data.

## Supporting information

S1 Text Detailed description of the results presented in Fig 3. (DOCX)

S1 Fig Temporal dynamics of CS in the *spoT* mutant. At low concentrations of NIT, growth inhibition establishes in the *spoT* mutant without delay. Panels A–D show equivalent information to Fig 6. Numerical data are available in S1 Data.
(TIF)

S2 Fig Temporal dynamics of CS in the *hemL* mutant. At low concentrations of NIT, growth inhibition establishes in the *hemL* mutant without delay. Panels A–D show equivalent information to Fig 6. Numerical data are available in S1 Data.
(TIF)

S3 Fig Summary of the main genes and mechanisms involved in CS to NIT. (TIF)

S1 Data Excel spreadsheet containing, in separate sheets, the underlying numerical data for all figure panels, the statistical analyses, and the proteomic data sets. (XLSX)

S1 Table DNA probes used for qRT-PCR. (XLSX)

S2 Table Strain list. (XLSX)

S3 Table DNA oligos used for cloning and recombineering. (XLSX)

## Abbreviations

Acs1: amilCP-cat-sacB cassette 1
Atox1: amilCP-toxin cassette 1
cfu: colony forming units
CPM: counts per minute
CS: collateral sensitivity
HCD: higher-energy collision dissociation
LB: lysogeny broth
MIC: minimum inhibitory concentration
NIT: nitrofurantoin
OD_600_: optical density at 600 nm
PBS: phosphate buffered saline
qRT-PCR: quantitative reverse transcription PCR
SulA: Suppressor of Lon
TB: tryptone broth
TEAB: triethylammonium bicarbonate
TMT: Tandem Mass Tag
