# Supplementary material for: Molecular mechanisms of collateral sensitivity to the antibiotic nitrofurantoin
Source: PLoS Biol. 2020 Jan 27;18(1):e3000612. doi: 10.1371/journal.pbio.3000612 (PMC7004380; doi:10.1371/journal.pbio.3000612)
Supplement: S1 Text — (DOCX) [file pbio.3000612.s001.docx]

**Effects of nitroreductase expression on NIT susceptibility.**

We measured the effects of single and dual overexpression of *nfsA* and *nfsB* from pBAD18 on the MIC of NIT relative to empty vector controls (EV). In the *S. enterica* wild type, the MIC was reduced substantially from 11 mg/l to 5.25 mg/l (2x), 6.75 mg/l (1.6x), or 2.6 mg/l (4.2x), by expression of the pBAD::*nfsA*, pBAD::*nfsB*, and pBAD::*nfsA-nfsB* constructs, respectively (Fig 3A, bars on the far right), indicating a potentially large contribution of nitroreductase expression for CS in the *hemL* mutant. In the *E. coli* wild type, however, expression of pBAD::*nfsA*, pBAD::*nfsB*, and pBAD::*nfsA-nfsB* hardly reduced the MIC (Fig 3A). The dual overexpression produced an MIC of 2.6 mg/l, i.e. a 1.4x reduction of MIC (Fig 3A). The observed small effects for the *E. coli* wild type could potentially be explained by a strong functional overlap of NfsA and NfsB in this species, where either protein can replace the activity of the other. To study the effect of overexpressing one nitroreductase in isolation, we constructed the scar-free deletion strains *∆nfsA*, *∆nfsB*, and the double knockout *∆nfsA ∆nfsB* in *E. coli*. The deletions produced moderate increases in MIC with ~1.5x and 2x increase in MIC for the vector-free *∆nfsA* (MIC = 8 mg/l) and *∆nfsB* (MIC = 5-6 mg/l) strains, respectively (Fig 4). The *∆nfsA ∆nfsB* strain had an epistatic MIC of 128 mg/l (32x increase), supporting a large functional overlap of the two nitroreductases. We then expressed the *E. coli* nitroreductases individually from the pBAD18 plasmid in strains where the other nitroreductase was deleted from the chromosome. Expression of pBAD::*nfsB* in *∆nfsA* reduced MIC from 7 mg/l to 4.2 mg/l, and expression of pBAD::*nfsA* in *∆nfsB* reduced MIC from 4.8 mg/l to 3.8 mg/l. The individual relative effects of nitroreductase expression therefore amount to roughly 2x and 1.3x for *nfsA* and *nfsB* in *E. coli* in our overexpression system. Complementation assays returned the MIC of the slightly resistant deletion strains to the wild type level (Fig 3A). Together, these data confirm a positive relation between nitroreductase expression and NIT susceptibility in our wild type strains. Furthermore, they indicate a larger contribution of nitroreductase expression for susceptibility of wild type strains of *S. enterica* (up to ~4.2x) compared to *E. coli* (up to ~1.4x).

To validate the effects of nitroreductase expression with a more sensitive assay than MIC, we measured the inhibitory effect of nitroreductase expression on growth rates at sub-MIC concentrations of NIT (i.e. at 1, 2, and 4 mg/l; Fig 3B). Previous work has shown that growth rate measurements in presence of low drug levels allow for detection of much smaller differences in susceptibility as compared to traditional MIC assays [1,2]. The growth rate data at the higher NIT concentrations was significantly correlated with the MIC measurements (Pearson’s correlation, divisions per h ~ MIC, *df* = 22; for growth at 4 mg/l NIT: *t* = 8.379, *P* = 2.715e-08, *R^2^* = 0.7614; for growth at 2 mg/l NIT: *t* = 4.398, *P* = 0.0002, *R^2^* = 0.4678; no significant correlation for growth at 1 mg/l). Overexpression of nitroreductase significantly reduced exponential growth relative to EV in all genetic backgrounds (FDR-adjusted P ≤ 0.00076, see S1 Data for detailed statistics results), with the strongest reductions occurring at the higher NIT concentrations and in *S. enterica* (Fig 3B). In some treatments, growth rates were mildly reduced compared to empty vector in the absence of arabinose, however the relative reductions were significantly increased in media with arabinose (FDR-adjusted P ≤ 0.0026, see S1 Data for detailed statistics results).

We quantified the importance of nitroreductase expression for the bactericidal activity of NIT by conducting time-kill experiments of *E. coli* at 24 mg/l (6x MIC of the *E. coli* wild type). The time-kill data were statistically analysed using a generalized linear mixed model (GLMM) and *post-hoc* tests (as described in S1 Data). We first measured the effect of chromosomal nitroreductase gene deletion on killing by NIT. The single deletion mutants initially showed increased survival, but the overall dynamics were not significantly different from the wild type, owing to substantial killing at the later time points (Fig 3C). The double mutant, however, was able to grow at 24 mg/l and showed significantly increased survival dynamics (FDR-adjusted *P* = 0.0211, see S1 Data for detailed statistics results) compared to the wild type and the single deletion strains. We then measured the nitroreductase overexpression effects on killing rate with the pBAD constructs (Fig 3D). Single overexpression of either nitroreductase and dual overexpression of both nitroreductases significantly reduced survival of *E. coli* at 24 mg/l (FDR-adjusted *P* = 6.14E-07 for pBAD::*nfsA* vs. EV, 0.00375 for pBAD::*nfsB* vs. EV, and 6.49E-07 for pBAD::*nfsA-nfsB* vs. EV). The single expression constructs produced distinct temporal dynamics. The killing curve of pBAD::*nfsA* corresponded to that of pBAD::*nfsA-nfsB* and showed biphasic dynamics with rapid killing in the first 2 h, followed by slower killing. This contrasted with the comparatively steady dynamics of pBAD::*nfsB* (Fig 3D). There was no difference in killing between treatments with and without arabinose induction. The full bactericidal activity of NIT thus appears to be conferred by leaky expression alone, whilst the bacteriostatic effect of NIT, as measured at the lower concentrations (Fig 3B) and by Etest (Fig 3A), increased with the presence of the inducer. Altogether, the conducted analyses (MIC, growth rate and time-kill) firmly establish a positive relation between nitroreductase expression and NIT susceptibility.

**References**

1. Chevereau G, Dravecká M, Batur T, Guvenek A, Ayhan DH, Toprak E, et al. Quantifying the Determinants of Evolutionary Dynamics Leading to Drug Resistance. PLOS Biol. 2015;13: e1002299. doi:10.1371/journal.pbio.1002299

2. Bergmiller T, Andersson AMC, Tomasek K, Balleza E, Kiviet DJ, Hauschild R, et al. Biased partitioning of the multidrug efflux pump AcrAB-TolC underlies long-lived phenotypic heterogeneity. Science. 2017;356: 311–315. doi:10.1126/science.aaf4762
