# Supplementary figures and images for: Molecular mechanisms of collateral sensitivity to the antibiotic nitrofurantoin
Source: PLoS Biol. 2020 Jan 27;18(1):e3000612. doi: 10.1371/journal.pbio.3000612 (PMC7004380; doi:10.1371/journal.pbio.3000612)

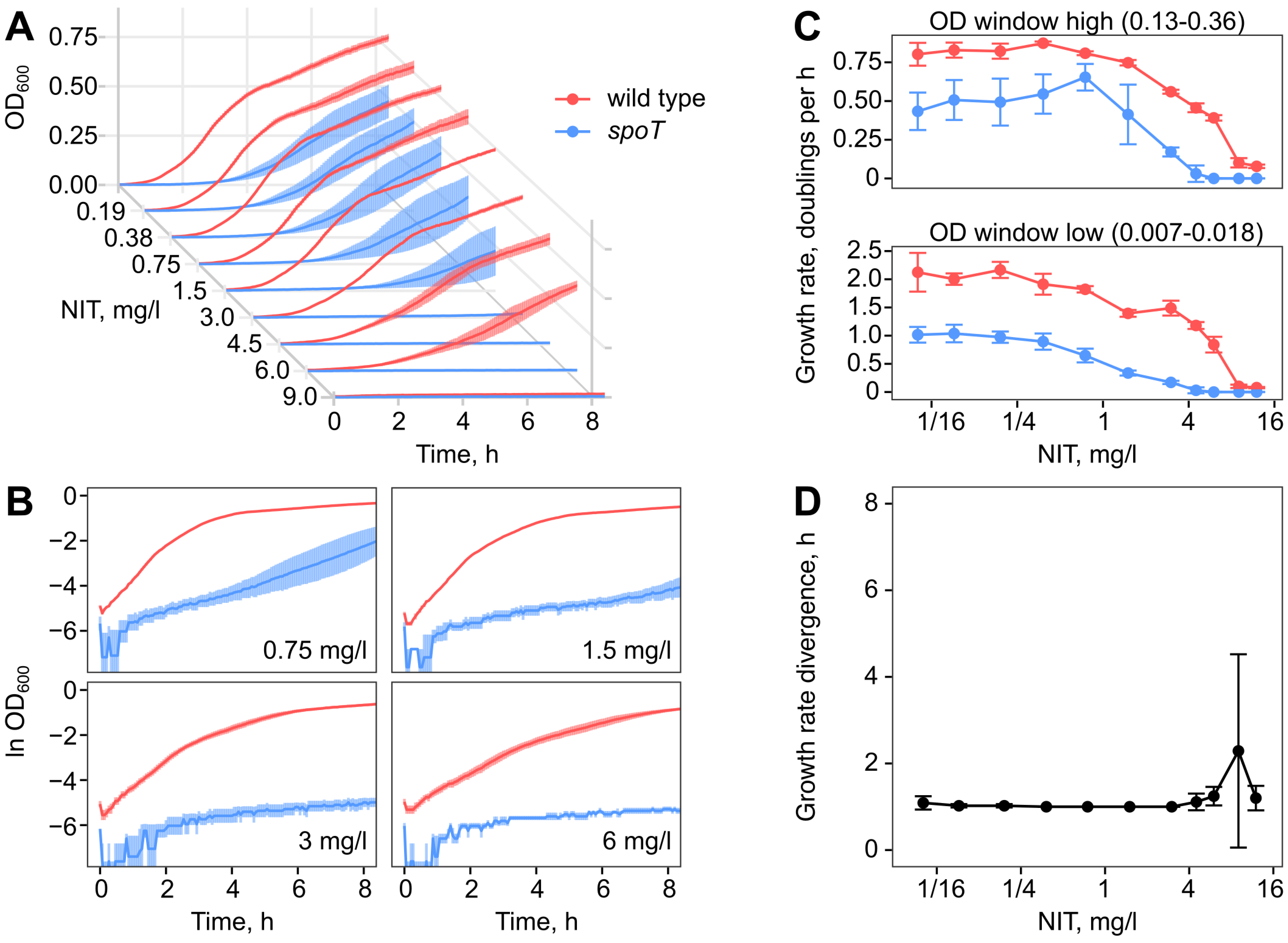

Supplement: S1 Fig — At low concentrations of NIT, growth inhibition establishes in the spoT mutant without delay. Panels A–D show equivalent information to Fig 6. Numerical data are available in S1 Data. (TIF) [file pbio.3000612.s002.tif]

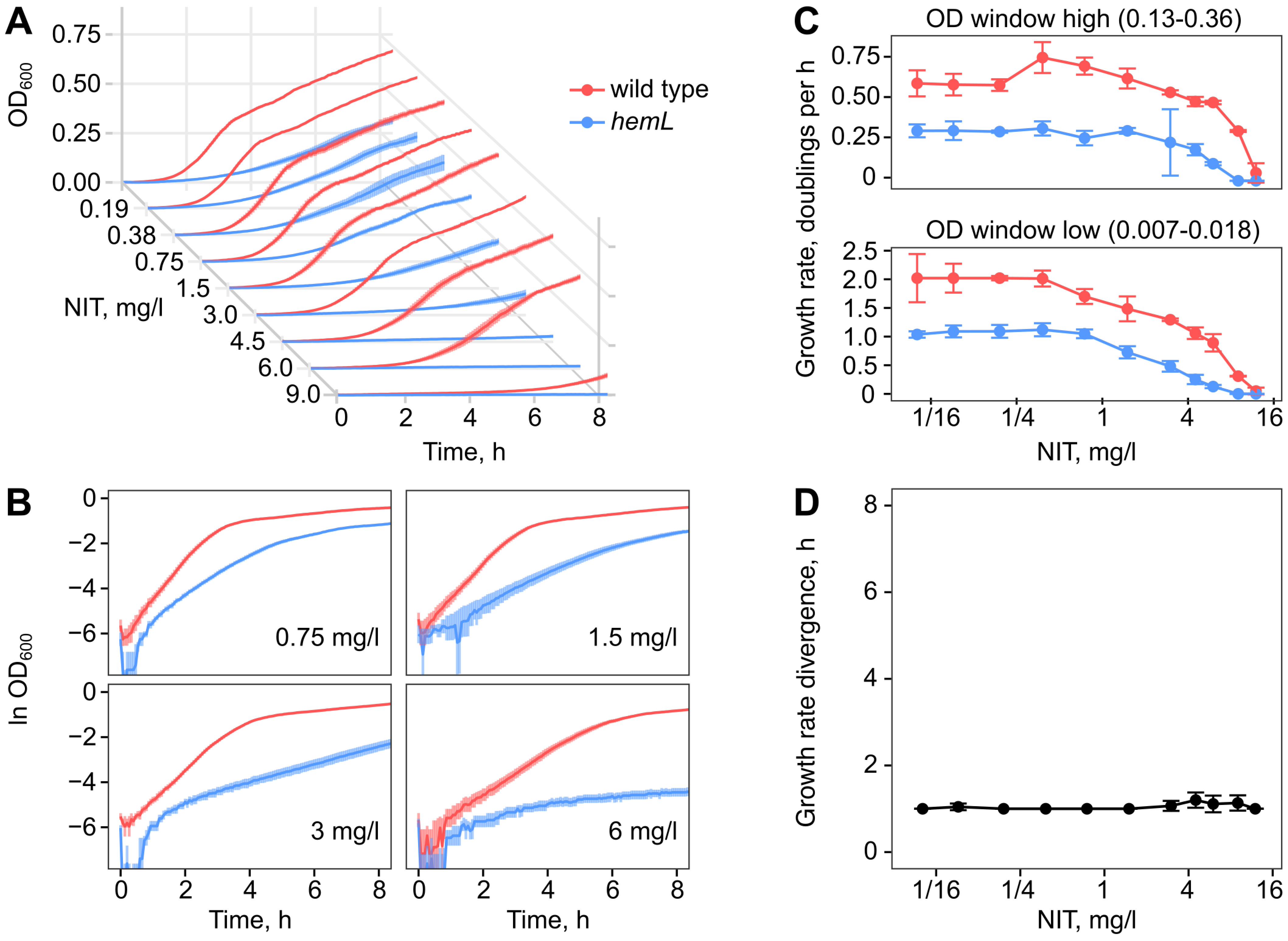

Supplement: S2 Fig — At low concentrations of NIT, growth inhibition establishes in the hemL mutant without delay. Panels A–D show equivalent information to Fig 6. Numerical data are available in S1 Data. (TIF) [file pbio.3000612.s003.tif]

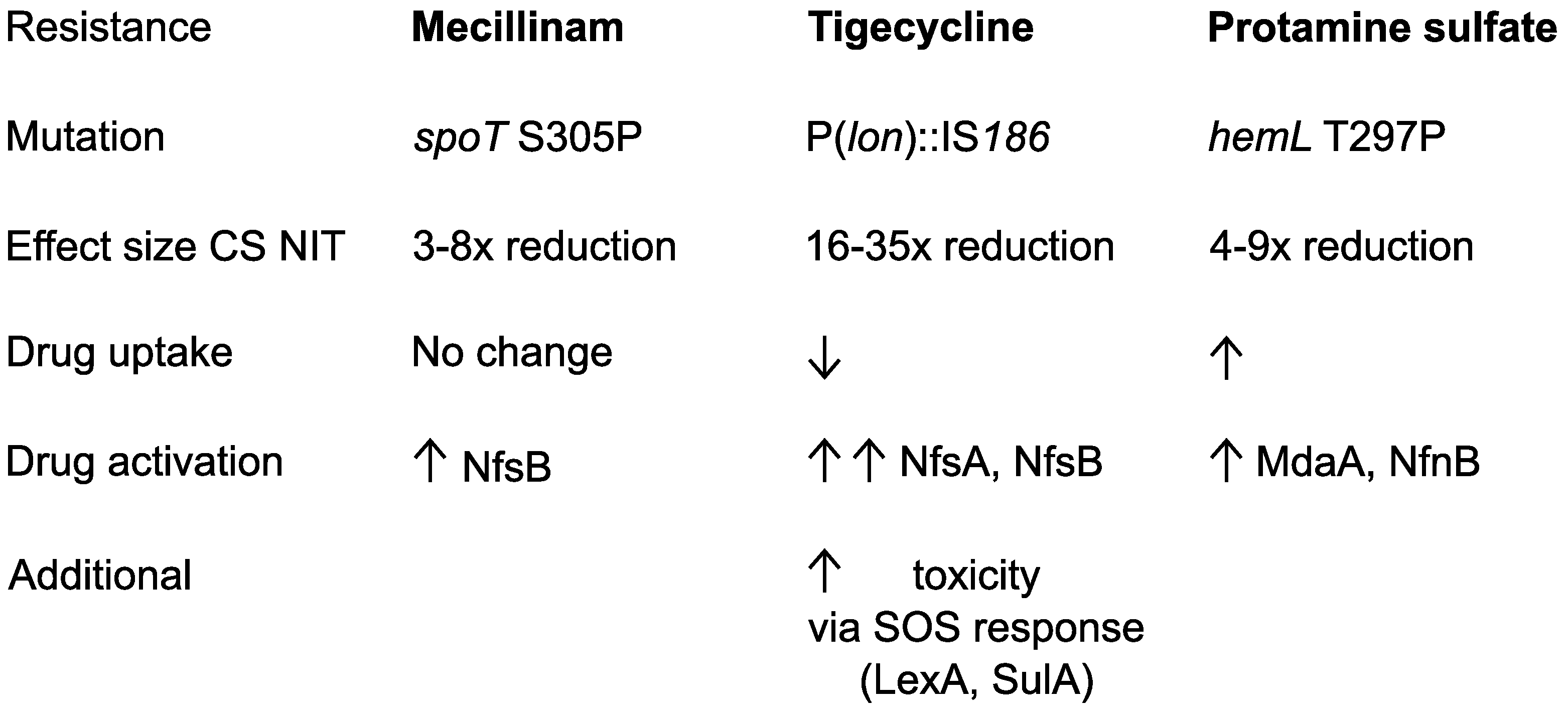

Supplement: S3 Fig — (TIF) [file pbio.3000612.s004.tif]
